# Cost-effectiveness analysis of Tumor Treating Fields treatment in Chinese patients with metastatic non-small cell lung cancer

**DOI:** 10.3389/fpubh.2024.1276049

**Published:** 2024-10-22

**Authors:** Zhengda Pei, Ningping Xiao, Pei Yang

**Affiliations:** ^1^Graduate Collaborative Training Base of Hunan Cancer Hospital, Hengyang Medical School, University of South China, Hengyang, China; ^2^Hunan Cancer Hospital, The Affiliate Hospital of Xiangya Medical School, Central South University, Changsha, China; ^3^Key Laboratory of Translational Radiation Oncology, Changsha, China

**Keywords:** metastatic non-small cell lung cancer (mNSCLC), Tumor Treating Field (TTFields), standard-of-care (SOC), immune checkpoint inhibitor (ICI), incremental cost-effectiveness ratios (ICERs)

## Abstract

**Background:**

The LUNAR trial demonstrated the significant efficacy and safety of Tumor Treating Fields (TTFields) plus standard-of-care (SOC) [immune checkpoint inhibitor (ICI) and docetaxel (DTX)] for patients with previously treated metastatic non-small cell lung cancer (mNSCLC). However, it remains uncertain as to whether the high costs are justified by the corresponding survival benefits. Here, the cost-effectiveness of using TTFields plus SOC for treating mNSCLC was evaluated from the perspective of the Chinese healthcare system.

**Methods:**

A Markov model with a 15-year time horizon was established and used to comparedeveloped to enable the simulation of treatment-associated costs and patient outcomes when comparing TTFields plus SOC to SOC alone. Primary outcomes for these analyses included total costs, life-years (LYs), quality-adjusted LYs (QALYs), and incremental cost-effectiveness ratio (ICER) values. The impact of paramere uncertainty on model outcomes was evaluated through sensitivity analyses. Additional subgroup and scenario analyses were also performed to extend these results.

**Results:**

While TTFields plus SOC exhibited a $74,688 increase in total costs relative to SOC ($96,092 vs. $21,404), it was associated with 0.38 additional QALYs (1.08 vs. 0.82 QALYs) for an ICER of $284,490/QALY. This value exceeded the $35,983/QALY willingness-to-pay (WTP) threshold selected for these analyses by a wide margin. Relative to ICI and DTX treatment, the incremental costs of TTFields plus ICI and TTFields plus DTX were $78,115 and $71,307, respectively, with corresponding gains of 0.42 and 0.13 QALYs, yielding ICERs of $187,434/QALY, and $546,386/QALY. The parameter that most strongly impacted the results of these analyses was the cost of TTFields.

**Conclusion:**

The results indicated that given current treatment costs, TTFields plus SOC was insufficiently cost-effective in treating patients with mNSCLC in China, although TTFields plus ICI yields substantial health benefits.

## 1 Introduction

Lung cancer remains the second most common and most deadly cancer type globally, with 2,206,771 diagnoses and 1,796,144 deaths in 2020 alone (1). The incidence and mortality of lung cancer are particularly high in China, with an estimated 816,000 diagnoses and 715,000 deaths annually (2). Lung cancer is broadly classified into small cell lung cancer (SCLC) and non-SCLC (NSCLC) subtypes, with NSCLC being the more common subtype and roughly 70% of patients with NSCLC first being diagnosed when their disease is already locally advanced or metastatic (3, 4). The 5-year survival of metastatic NSCLC (mNSCLC) patients is only 26% (3, 4). Chemotherapy-based has been considered the standard treatment for mNSCLC (5–9). However, the efficacy of this approach remains poor and the safety profile is unsatisfactory, with patients experiencing an average overall survival (OS) of 10–14 months (5–8). There is thus a clear need to develop new treatment strategies that can confer prognostic benefits to individuals with mNSCLC.

While the recent development of immune checkpoint inhibitor (ICI) therapies has prolonged the survival of patients with certain cancers, second-line ICI administration is only associated with an average 14-month survival interval (9–11), emphasizing a need for additional innovative treatment options. The Tumor Treating Fields (TTFields) technology was recently designed as a unique approach to solid tumor treatment (12). This non-invasive therapy entails the localized delivery of alternating low-intensity, mid-frequency (100–300 kHz) electric fields to the target (12, 13). The generation of these uneven electrical fields within cancer cells can adversely impact macromolecules and organelles during the process of cellular division such that abnormal chromosomal segregation and multinucleation are more likely to occur, impacting subsequent daughter cell replication (12). The efficacy of TTFields plus standard-of-care (SOC) for patients with mNSCLC were confirmed in the phase III LUNAR (NCT02973789) trial. This study revealed that TTFields plus SOC significantly prolonged PFS and OS (14). These promising survival outcomes emphasize the potential value of this approach to mNSCLC management such that there may be sufficient justification to recommend its inclusion in international guidelines and its widespread clinical deployment.

While the combination of TTFields with SOC (ICI and DTX) yielded promising efficacy when used for mNSCLC patient management, the costs of this novel treatment regimen are substantial, about $9,355 per month, which is much higher than average GDP in China, imposing a substantial economic burden on affected patients and the national healthcare system. In resource-limited nations such as China, pharmacoeconomic analyses can provide valuable insights that can guide the rational allocation of limited medical resources by policymakers. This study was thus developed with the goal of evaluating the cost-effectiveness of TTFields plus SOC (ICI and DTX) as an approach to treating patients with mNSCLC from the Chinese healthcare system perspective.

## 2 Materials and methods

The Consolidated Health Economic Evaluation Reporting Standards 2022 (CHEERS 2022) checklist designed by the International Society for Pharmacoeconomics and Outcomes Research (ISPOR) was used when performing the present study (15) (Supplementary Table S1). All key data used to conduct these analyses were extracted from the LUNAR trial and NovoCure Ltd. (14).

### 2.1 Patients and interventions

For this study, a theoretical population of 276 mNSCLC patients with disease progression during or following platinum therapy (prior ICI permitted) (14). Of these patients, 137 (49.6%) and 139 (56.4%) respectively underwent treatment with TTFields (150 kHz ≥8 h/d) plus SOC (ICI using 1:1:1 ratios of 200 mg of pembrolizumab, 360 mg of nivolumab, and 1,200 mg of atezolizumab, as well as 75 mg/m2 of DTX, given every 3 weeks) or SOC treatment (9, 14) (Supplementary Table S2). SOC treatment-related decisionmaking was conducted by assuming all patients were 65-year-old males weighing 65 kg, with a height of 164 cm, and a 1.72 m^2^ body surface area (16). Following these treatments, 18.0 and 26.0% of patients, respevtively, exhibited progressive disease (PD) and were administered the best supportive care (BSC) as per the design and guidelines of the study (9, 14). All patients received terminal care before treatment-related death (9, 14).

### 2.2 Model establishment

The simulation of mNSCLC patient clinical and economic outcomes for this study was performed with a Markov model implemented in TreeAge Pro 2022. Disease progression was simulated using three states (PFS, PD, death) that were mutually exclusive (Supplementary Figure S1). The patients were all classified as PFS in the beginning and every 6 weeks their status could change to either PD or death, with PD patients also able to shift to death status. The time horizon for this model was 15 years. Primary model outcome measures included total costs, life years (LYs), quality-adjusted life-years (QALYs), and incremental cost-effectiveness ratio (ICER) values. These results were evaluated in light of a willingness-to-pay (WTP) threshold of $35,983/QALY which was equal to three times the GDP *per capita* of China in 2022. A 5% annual discounting rate was applied to all costs and utility values based on the World Health Organization (WHO) and China Pharmacoeconomic Evaluation Guidelines (16–18).

Transition probability values were calculated based on the extraction of short-term OS and PFS Kaplan–Meier curve data and the extrapolation thereof using GetData Graphics Digitizer (v 2.26), Matlab (v R2020a), and R Studio (v 4.2.2). Survival curve parameter distributions were evaluated for fit to the Gompertz, Weibull, Exponential, Log-normal, and Log-logistic distributions, with a combination of visual inspection, the Akaike Information Criterion, and the Bayesian Information Criterion being used to evaluate the goodness of fit (Supplementary Figure S2; Supplementary Table S3) (16). This approach led to the selection of the Weibull distribution such that the following formula was used to calculate the transition probabilities over time:


$$\left(1-\exp\left\{\lambda {\left(t-u\right)}^{\gamma }-\lambda t^{\gamma }\right\}\right)$$

Where *u* denotes the Markov period, *t* denotes the current model period, and *λ* and *γ, respectively,* correspond to the scale and shape parameters (16).

### 2.3 Utility and cost inputs

As the LUNAR trial did not collect data related to patient quality of life (QoL), a utility value of 0.804 was assigned to the PFS state and a value of 0.321 to the PD state, according to previous publications (19). Reductions in these utility value were based on the disutility of adverse events (AEs) reported in previous researchs and their corresponding probability in LUNAR trial (Table 1) (20–22).

**Table 1:** Clinical and health parameters.

Variable	Baseline value (Range)	Reference	Distribution
Clinical parameters			
Weibull survival model for OS			
TTFields plus SOC	Scale = 0.059090, Shape = 0.927193	(14)	NA
SOC	Scale = 0.059479, Shape = 1.031140	(14)	NA
TTFields plus ICI	Scale = 0.055872, Shape = 0.861250	(14)	NA
ICI	Scale = 0.050078, Shape = 1.040615	(14)	NA
TTFields plus DTX	Scale = 0.051529, Shape = 1.096903	(14)	NA
DTX	Scale = 0.068828, Shape = 1.054559	(14)	NA
Weibull survival model for PFS			
TTFields plus SOC	Scale = 0.185990, Shape = 0.856320	(14)	NA
SOC	Scale = 0.234310, Shape = 0.805790	(14)	NA
Rate of post-discontinuation therapy			
TTFields plus SOC	0.18 (0.14–0.22)	(14)	Beta
SOC	0.26 (0.21–0.30)	(14)	Beta
Risk for main AEs in TTFields plus SOC			
Dyspnea	0.07 (0.06–0.08)	(14)	Beta
Anemia	0.08 (0.06–0.10)	(14)	Beta
Pneumonia	0.11 (0.09–0.13)	(14)	Beta
WBC count decreased	0.14 (0.11–0.17)	(14)	Beta
Risk for main AEs in SOC			
Anemia	0.08 (0.06–0.10)	(14)	Beta
Fatigue	0.08 (0.06–0.10)	(14)	Beta
Pneumonia	0.11 (0.09–0.13)	(14)	Beta
WBC count decreased	0.14 (0.11–0.17)	(14)	Beta
Health parameters			
Utility and disutility			
Utility of PFS	0.804 (0.643–0.965)	(19)	Beta
Utility of PD	0.321 (0.257–0.385)	(19)	Beta
Disutility of anemia	0.073 (0.058–0.088)	(20–22)	Beta
Disutility of WBC count decreased	0.090 (0.072–0.108)	(20–22)	Beta
Disutility of pneumonia	0.090 (0.072–0.108)	(20–22)	Beta
Disutility of fatigue	0.751 (0.601–0.901)	(20–22)	Beta
Disutility of dyspnea	NA	NA	NA
Discount rate	0.05 (0–0.08)	(16–18)	Uniform

OS, overall survival; PFS, progression-free survival; TTFields, tumor treating field; SOC, standard of care; ICI, immune checkpoint inhibitor; DTX, docetaxel; WBC, white blood cell; AEs, adverse events; PD, progressive disease; NA, not applicable.

Only direct medical costs were taken into consideration as the study was performed from the perspective of Chinese healthcare systems. Including the costs of treatment, tumor imaging, laboratory tests, severe treatment-related AE management, administration, BSC, and terminal care (Table 2). Real-world data and information from NovoCure Ltd. were used to establish treatment-related costs, while all other cost data were derived from a previous publication (16, 23, 24). Costs were reported in dollars converted using the June 2023 exchange rate (*$1 = 7.1417*).

**Table 2:** Cost estimates.

Parameters	Baseline value (Range)	Reference	Distribution
Treatment cost, $			
TTFields per month	9,355 (7,484–11,226)	NovoCure Ltd.	Gamma
Pembrolizumab per cycle	6,691 (5,178–7,766)	Locoal	Gamma
Nivolumab per cycle	6,472 (17,543–26,315)	Locoal	Gamma
Atezolizumab per cycle	5,249 (4,199–6,299)	Locoal	Gamma
Docetaxel per cycle	41 (33–49)	Locoal	Gamma
Cost of AEs			
TTFields plus SOC	302 (242–362)	(16, 23, 24)	Gamma
SOC	310 (248–372)	(16, 23, 24)	Gamma
Administration per cycle	34 (27–41)	(16)	Gamma
Best supportive care per cycle	209 (167–251)	(16)	Gamma
Laboratory and tumor imaging per cycle	637 (510–764)	(16)	Gamma
Terminal care per patient	2,102 (1,682-2,522)	(16)	Gamma

TTFields, tumor treating field; SOC, standard of care.

### 2.4 Sensitivity analyses

To confirm model stability and robustness, one-way and probabilistic sensitivity analyses were conducted. In one-way sensitivity analyses, over 20 key parameters were varied within ±20% of base values in a tornado diagram (16, 18). For probabilistic analyses, 1,000 Monte Carlo simulations were used to randomly sample the distributions for all parameters, with results being presented using cost-effectiveness acceptability curves and scatter plots (16, 18).

### 2.5 Subgroup and scenario analyses

Subgroup analyses were conducted for different pathologic types reported in the LUNAR trial [squamous cell carcinoma (SCC) and non-squamous cell carcinoma (NSCC)], although only survival curves and mOS data (13.9 and 12.6 months) were available for these subgroups (14). All other information was thus assumed to be the same as that for the overall patient population as per a previous approach (20).

Pharmacoeconomic study results can be used as a reference when pricing costly therapies and promoting their more widespread clinical application. At present TTFields is not covered by Medicare. To assess the potential implications of future reductions in TTFields costs, ICER values were recalculated as above assuming a 5, 10%, or 50% TTFields price reduction.

## 3 Results

### 3.1 Baseline results

Initial analyses revealed that the total costs of TTFields plus SOC, TTFields plus ICI, and TTFields plus DTX were $96,092, $100,700, and $91,145, respectively, generating 1.08, 1.32, and 0.84 QALYs. The corresponding costs of SOC, ICI, and DTX were $74,688, $78,115, and $71,307, respectively, generating 0.82, 0.90, and 0.71 QALYs. Based on these values, these three TTFields combination regimens exhibited corresponding ICERs of $284,490/QALY, $187,434/QALY, and $546,386/QALY. As these ICERs were substantially higher than the selected WTP threshold ($35,983/QALY), this suggests that TTFields plus SOC (ICI or DTX) is not a cost-effective treatment for patient with mNSCLC. The life expectancy gains for patients when comparing TTFields plus SOC vs. SOC, TTFields plus ICI vs. ICI, and TTFields plus DTX vs. DTX were 0.67 LYs (8.04 months), 1.15 LYs (13.80 months), and 0.26 LYs (3.12 months), respectively (Table 3).

**Table 3:** Results of the base-case analysis.

Treatment	Total cost, $	Overall LYs	Overall QALYs	ICER, $	
				per LY	per QALY
SOC	21,404	1.76	0.82	Reference	Reference
TTFields plus SOC	96,092	2.43	1.08	111,226	284,490
ICI	22,585	1.99	0.90	Reference	Reference
TTFields plus ICI	100,700	3.14	1.32	68,023	187,434
DTX	19,838	1.46	0.71	Reference	Reference
TTFields plus DTX	91,145	1.71	0.84	277,003	546,386

LYs, life-years; QALYs, quality-adjusted life-years; ICER, incremental cost-effectiveness ratio; TTFields, tumor treating field; SOC, standard of care; ICI, immune checkpoint inhibitor; DTX, docetaxel.

### 3.2 Sensitivity analysis results

**Figure 1:**
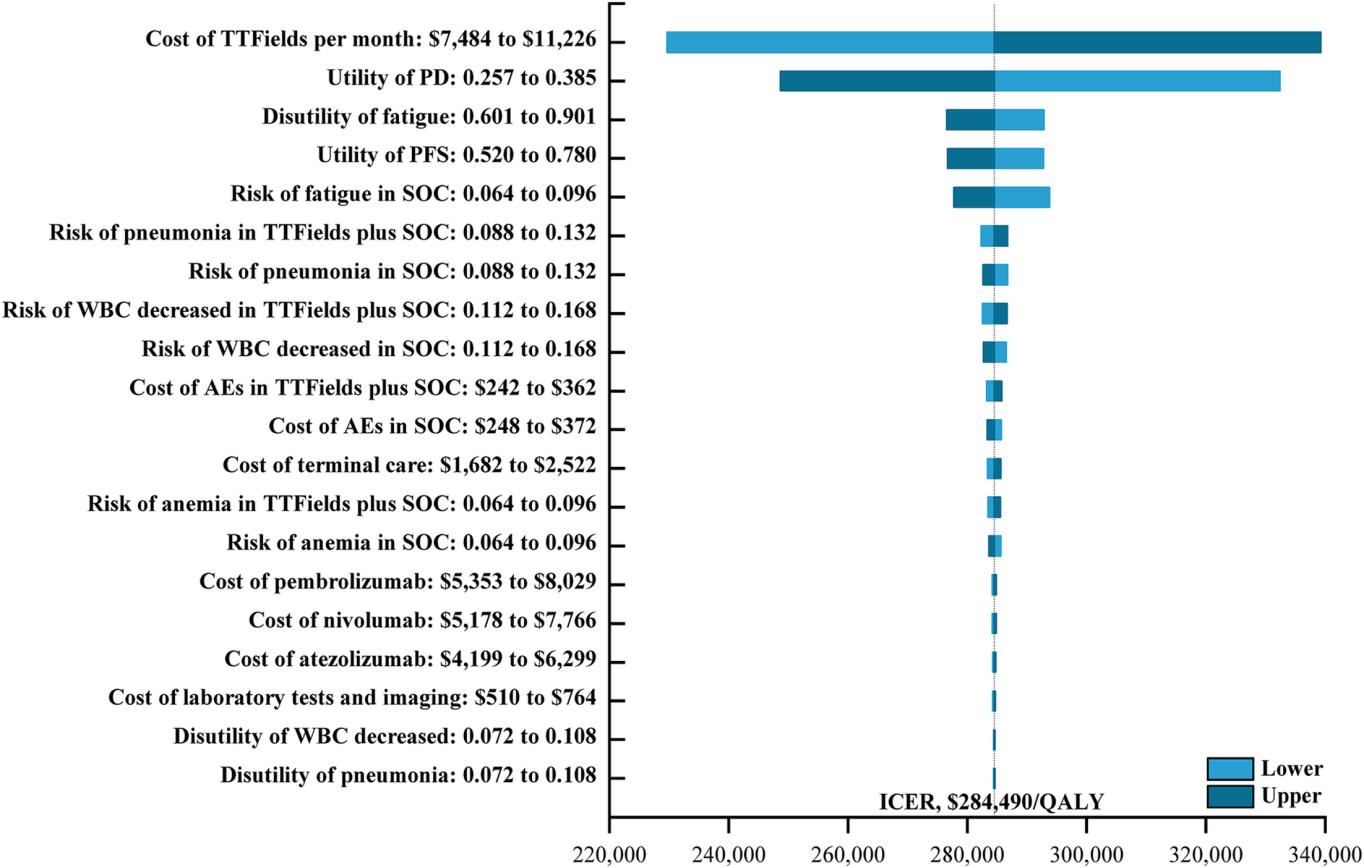
The one-way sensitivity analyses for the TTFields plus SOC versus the SOC. TTFields, tumor treating field; PD, progressive disease; PFS, progression-free survival; SOC, the standard-of-care; WBC, white blood cell; AEs, adverse events; ICER, incremental cost-effectiveness ratio; QALY, quality-adjusted life-year.

Univariate sensitivity analyses were also conducted (Figure 1), with tornado diagrams demonstrating that the cost of TTFields, the utility of PD, the disutility of fatigue, and the utility of PFS had the greatest impact on model outcomes. Other factors such as the costs of drug administration, DTX, or BSC largely failed to impact this model. ICER values did not exceed the established WTP threshold even when these parameters were changed, however, indicating that the results of the base-case analysis were robust.

**Figure 2:**
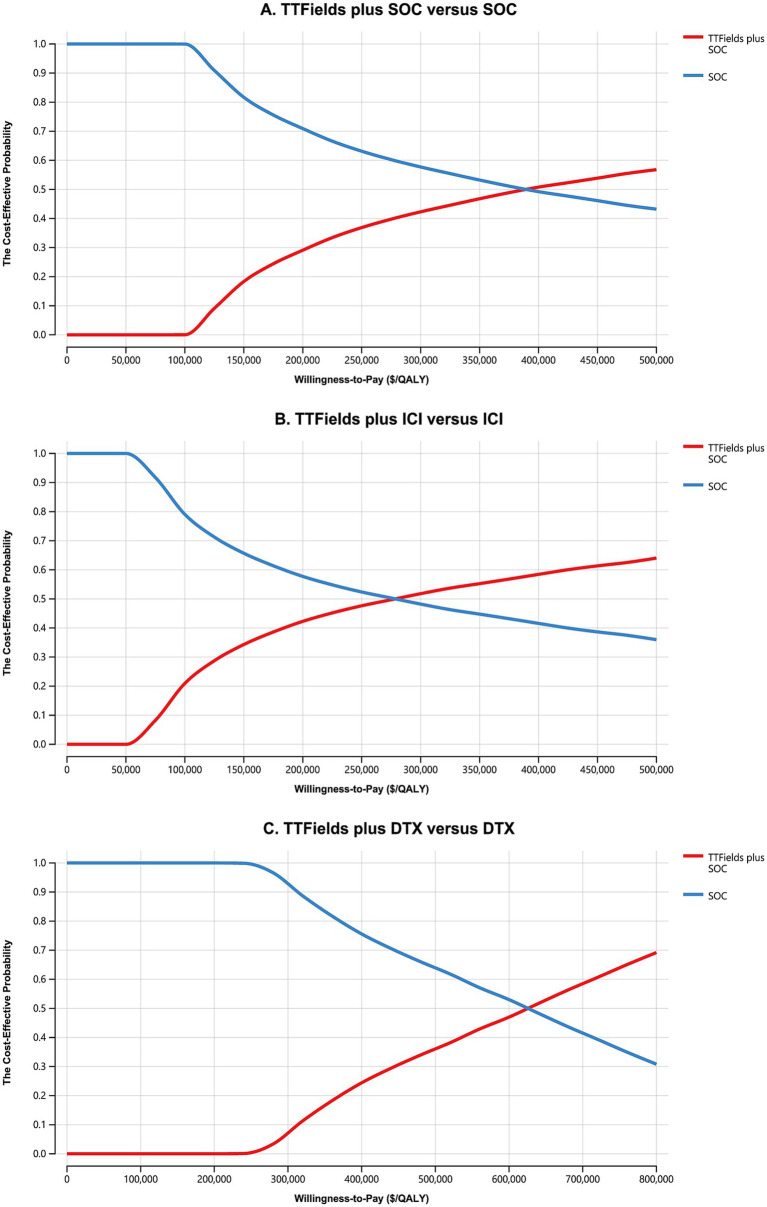
The cost-effectiveness acceptability curves for the TTFields plus SOC versus the SOC **(A)**, the TTFields plus ICI versus the ICI **(B)**, and the TTFields plus DTX versus the DTX **(C)**. TTFields, tumor treating field; SOC, the standard of care; QALY, quality-adjusted life-year; ICI, immune checkpoint inhibitor; DTX, docetaxel.

Cost-effectiveness acceptability curves indicated that TTFields plus SOC, TTFields plus ICI, and TTFields plus DTX were first cost-effective at respective WTP thresholds of $290,000/QALY, $190,000/QALY, and $550,000/QALY (Figure 2). When these WTP thresholds were increased to $400,000/QALY, $285,000, and $630,000/QALY, the TTFields plus SOC, TTFields plus ICI, and TTFields plus DTX regimens exhibited a > 50% chance of being cost-effective (Figure 2). Results from all 10,000 simulations fell above the WTP threshold (Supplementary Figure S3), revealing that TTFields plus SOC (ICI or DTX) is not cost-effective.

### 3.3 Subgroup and scenario analysis results

Subgroup analyses indicated that in SCC patients, TTFields plus SOC yielded an additional 0.26 QALYs at an incremental cost of $72,433 relative to SOC for an ICER of $273,741/QALY. For NSCC patients, TTFields plus SOC yielded an additional 0.18 QALYs at an incremental cost of $73,484 for an ICER of $410,594/QALY. As such, combined TTFields plus SOC treatment is not currently a cost-effective option for either of these pathologic types of mNSCLC (Supplementary Table S5).

With 5, 10, and 50% reductions in the cost of TTFields, the incremental costs (incremental benefits) of TTFields plus SOC versus SOC were $6,379 (0.26 QALYs), $9,974 (0.26 QALYs), and $38,736 (0.26 QALYs), respectively, with corresponding ICERs of $65,262.67/QALY, $37,991/QALY, and $147,546/QALY. Similarly, the incremental costs (incremental benefits) of TTFields plus ICI versus ICI were $7,482 (0.42 QALYs), $11,200 (0.42 QALYs), and $40,940 (0.42 QALYs), respectively, with corresponding ICERs of $17,953/QALY, $26,873/QALY, and $98,233/QALY, while the incremental costs (incremental benefits) of TTFields plus DTX versus DTX were $5,534 (0.13 QALYs), $8,996 (0.13 QALYs), and $36,690 (0.13 QALYs), respectively, with corresponding ICERs of $42,405/QALY, $68,930/QALY, and $281,133/QALY, (Supplementary Table S5).

## 4 Discussion

TTFields has achieved significant effacy in the treatment of newly diagnosed Glioblastoma and relapsed glioblastoma in Chinese population which has been recommended by the Chinese Society of Clinical Oncology (CSCO) and the National Comprehensive Cancer Network (NCCN) guidelines, and has been covered by medical insurance in some areas of China (25–27). Currently, clinical studies of TTFields are being carried out in China for pancreatic cancer, unresectable gastroesophageal junction or gastric adenocarcinoma, brain metastases, and so on. Fortunately, TTFields has also achieved promising efficacy in NSCLC, but they have also incurred heavy costs such that patients, their families, and national healthcare systems have had to shoulder a heavy financial burden. In 2017, the total estimated economic burden of lung cancer in China was estimated at $25,069 million, over 55% of which was attributable to direct medical spending ($13,971 million), and this number is forecast to balloon to $40.4 billion by 2025 and $53.4 billion by 2030 (28). Health economics analyses have emerged as important tools capable of guiding national medical insurance negotiation efforts. By assessing the cost-effectiveness of a promising treatment strategy associated with clear clinical benefits, it is possible to provide direct evidence that can inform rational treatment planning and insurance payment-related decision-making, ensuring sustainable health system development. Previous published studies are both the cost-effectiveness analysis of TTFields from the perspective of the United States (29, 30). However, the cost of treatment and the cost of treatment for adverse events vary in different regions, which can affect the cost-effectiveness of treatment options. This research is the first to use a three-state Markov model to evaluate the cost-effectiveness of TTFields plus SOC (ICI and DTX) as a treatment for patients with mNSCLC from the perspective of China.

The base-case analysis revealed that TTFields plus SOC (ICI and DTX) was not a cost-effective treatment compared to SOC (ICI and DTX) at the $35,983/QALY WTP threshold, with an ICER of $284,490/QALY ($187,434/QALY and $546,386/QALY). Probabilistic sensitivity analyses and acceptability curves further confirmed that this combination regimen had a 0% likelihood of cost-effectiveness at current pricing levels. Although TTFields plus SOC (ICI and DTX) is not an economically justified treatment strategy in China, this does not indicate that patients should be administered therapies with poorer efficacy. In one-way sensitivity analyses, the cost of TTFields was found to significantly affect model outcomes. When the monthly costs of TTFields were reduced from $9,355 to $467.75, and $935.50 in scenario analyses, TTFields plus SOC and TTFields plus ICI were found to be potentially cost-effective, whereas TTFields plus DTX was never cost-effective irrespective of these changes in TTFields pricing. Under the 13th Five-Year Plan for the Chinese healthcare system, the government has launched a centralized drug procurement program and medical insurance reimbursement policy (31, 32). This has coincided with some efforts to reduce anticancer treatment costs, with many having been reduced significantly in price following negotiation and entry into the Chinese market (31, 32). Further changes in the price of this innovative therapeutic approach thus offer the potential for TTFields to be cost-effective in the future following its entry into the Chinese procurement list. The present data provide a valuable resource to guide clinical application, guideline establishment, and reimbursement strategies for TTFields treatment.

From the economic evaluation of novel antitumor treatments frequently reveal that these therapies are not cost-effective upon their initial market entry, even if costs were reduced by over 90%. There are several potential explanations for this phenomenon in the present case. Despite the high costs associated with TTFields-based regimens, the survival benefits attained by treated patients are relatively small such that ICER values are large. These results are consistent with findings from other studies that have consistently indicated that TTFields-based treatment approaches generally yield an incremental survival benefit of less than 1 QALY despite incremental costs ranging from $188,637 to $496,827, such that the associated ICERs exceed established WTP thresholds (33–35). The precise WTP threshold value selected for a given analysis has a major impact on the degree to which a given therapy is cost-effective. The WHO recommends a WTP threshold equal to a value from 3 times the GDP *per capita*, prompting the selection of $35,983/QALY as the threshold for China in the present study (36, 37). These proposed guidelines, however, have been controversial. In high-income nations such as the USA where the GDP *per capita* is $76,400, this can yield a WTP threshold of $229,200/QALY, whereas in the UK and China the respective GDP values of $45,900 and $11,994, the corresponding WTP thresholds are $137,700/QALY and $35,983/QALY. In low-income nations, however, these guidelines can be very restrictive such that in Ethiopia, for example, the WTP threshold would be as high as $3,082.8/QALY based on a GDP *per capita* of $1,027.6. This approach thus markedly limits the accessibility of novel anticancer treatments to certain countries, exacerbating healthcare inequality are raising ethical concerns. As such, it is important to assess the cost-effectiveness of a given regimen in light of the costs and corresponding WTP threshold values specific to a given country. In addition to the price of a drug, the efficacy of a drug also affects whether it is cost-effective. When the WTP threshold are high enough, a modest improvement in survival can make a given drug cost-effective. In contrast, at low WTP threshold, a more substantial survival benefit is needed for a given drug to be cost-effective. We further performed sensitivity analyses by adjusting the utility values. The ICER was $275,061/QALY to $284,689/QALY when the utility value of PFS in the model was adjusted from 0.8 to 1. When utility of PD was adjusted from 0.32 to 0, ICER was $285,130/QALY to $1,016,903/QALY. However, this does not change the conclusions. Therefore, when a new drug is introduced into a developing country, it needs to have better efficacy and a lower price to make it cost effective in developing countries.

The present study included analyses for mNSCLC patients in specific pathological subgroups. Although TTFields plus SOC was associated with greater benefits in SCC patients as compared to NSCC patients, it was not a cost-effective regimen in either subgroup, extending patient survival by just 0.26 LYs (3.12 months) and 0.18 LYs (2.16 months) in individuals with SCC and NSCC, respectively, relative to SOC alone. This, coupled with respective incremental costs of $72,433 and $73,484, yielded ICER values in excess of the established WTP threshold. These efficacy outcomes are also consistent with prior data highlighting differences in the composition of the tumor immune microenvironment in SCC and NSCC patients and emphasizing the better survival outcomes in the former cancer type (38–42). In their pooled analyses of 43,808 NSCLC patients, Brambilla et al. found that the OS of SCC patients (HR, 0.62; 95% CI, 0.42 to 0.93) tended to be longer than that of NSCC patients (HR, 0.69; 95% CI, 0.40 to 1.19) (39). When reviewing studies published over the past 25 years, Hirsch et al. observed inconsistencies in reported data such that some results were more favorable for SCC patients whereas others were more favorable for NSCC or adenocarcinoma patients (42). While pronounced differences in the design of these studies and their analytical techniques make it impossible to firmly establish the prognostic relevance of tumor histology, there have been several reports to date emphasizing an association between histological subtypes and clinical outcomes (42). Given the ongoing development of novel innovative treatment strategies and improvements in histological classification strategies, further research focused on the association between tumor histology and therapeutic outcomes may help prolong NSCLC patient survival while enhancing the cost-effectiveness of new therapeutic protocols.

There are also limitations to this study. Primarily, as the LUNAR trial did not report relevant data of interest outside of the follow-up period, parameter distribution fitting was herein used to calculate survival data. Such extrapolation is inevitable but does contribute to greater model uncertainty. Secondly, there have been no reports to date focused on the health utility values associated with the TTFields-based treatment of NSCLC patients, and the utility values used herein were based on other therapeutic approaches such that they have the potential to bias the resultant data. Third, model simplification was achieved by only taking into account treatment costs and the costs of managing ≥3 grade AEs affecting >5% of patients. Moreover, immune-related AEs and adverse device events were also excluded from these analyses such that the cost estimates may be inaccurate. Even so, one-way sensitivity analyses suggested that AE management-related costs had a relatively minimal impact on model outcomes. Fourth, the follow-up protocols for patients in this analysis were proscribed on a standardized basis, whereas in a real-world setting, physicians will make individualized decisions regarding patient follow-up in light of details regarding disease progression and other factors. Lastly, the LUNAR trial did not provide PFS curves for patients from the overall population who underwent TTFields plus ICI or DTX therapy, or for those in the SCC and NSCC subgroups who underwent Tfields+SOC treatment. The associated results should thus be interpreted with caution. Even in light of these limitations, the present results offer value as a basis for evaluating TTFields plus SOC (ICI and DTX) as an approach to mNSCLC patient management.

## 5 Conclusion

In summary, the costs associated with second-line TTFields plus SOC (ICI and DTX) as an approach to treating patients with mNSCLC was herein assessed from a Chinese payer perspective. Although TTFields plus SOC was associated with better survival outcomes for treated patients, the associated costs strongly outweigh these benefits when using a WTP threshold, three times the 2022 Chinese GDP *per capita*. These findings can serve as a valuable reference to guide the care of patients with mNSCLC and the formulation of appropriate healthcare policies in China and other nations throughout Asia in the near future.

## Data Availability

The original contributions presented in the study are included in the article/Supplementary material, further inquiries can be directed to the corresponding author.
