# Supplementary material for: Cost-effectiveness analysis of Tumor Treating Fields treatment in Chinese patients with metastatic non-small cell lung cancer
Source: Front Public Health. 2024 Oct 22;12:1276049. doi: 10.3389/fpubh.2024.1276049 (PMC11534589; doi:10.3389/fpubh.2024.1276049)
Supplement: Supplementary file 1 [file Data_Sheet_1.docx]

**Figure S1.** Model Structure.

**Figure S2.** Kaplan-Meier Curve Fitting and Extrapolation.

**Figure S3.** Probability Sensitivity Analysis Scatter Plot.

**Table S1.** The CHEERS 2022 checklist.

**Table S2.** Details of Treatment Strategy and Unit Costs.

**Table S3.** Summary of Statistical Goodness-of-fit of K-M Curve.

**Table S4.** Results of Subgroup Analyses.

**Table S5.** Discounting and Cost-Effectiveness Results.

**Figure S1. Model Structure.**


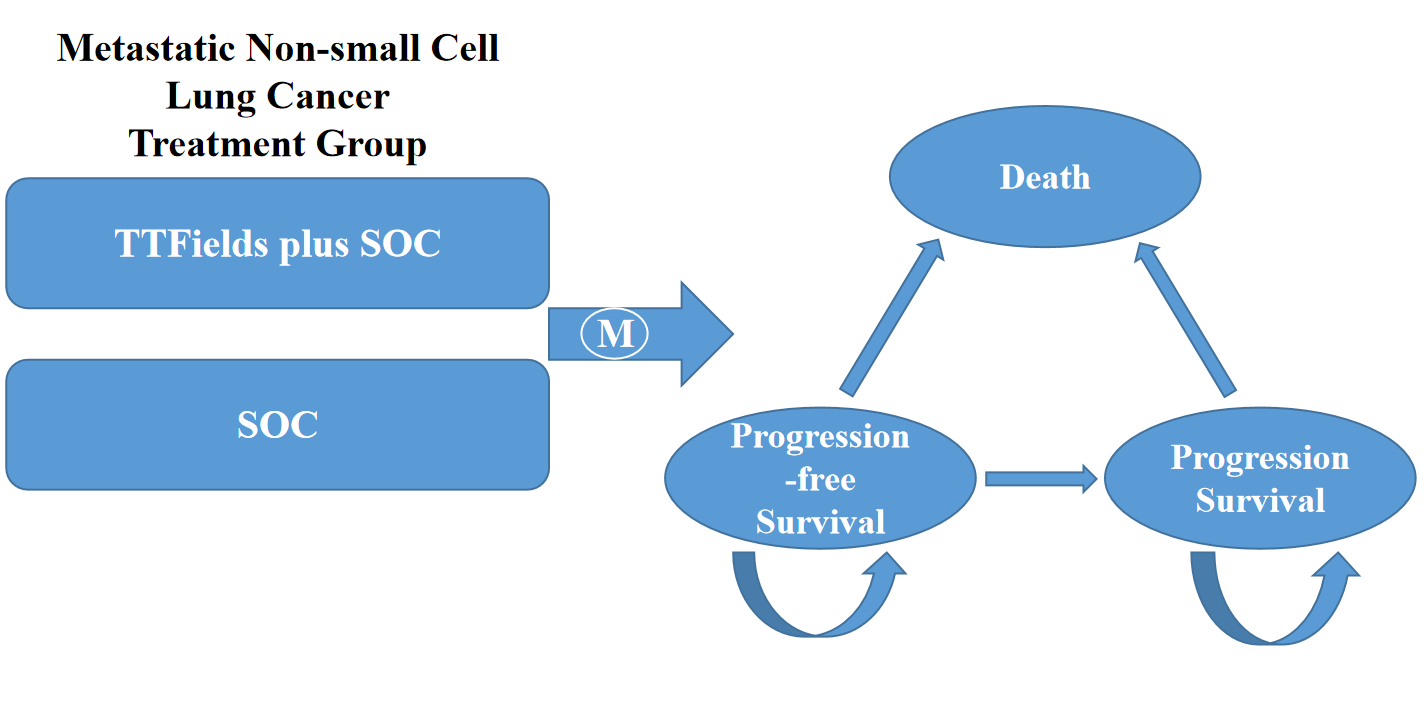


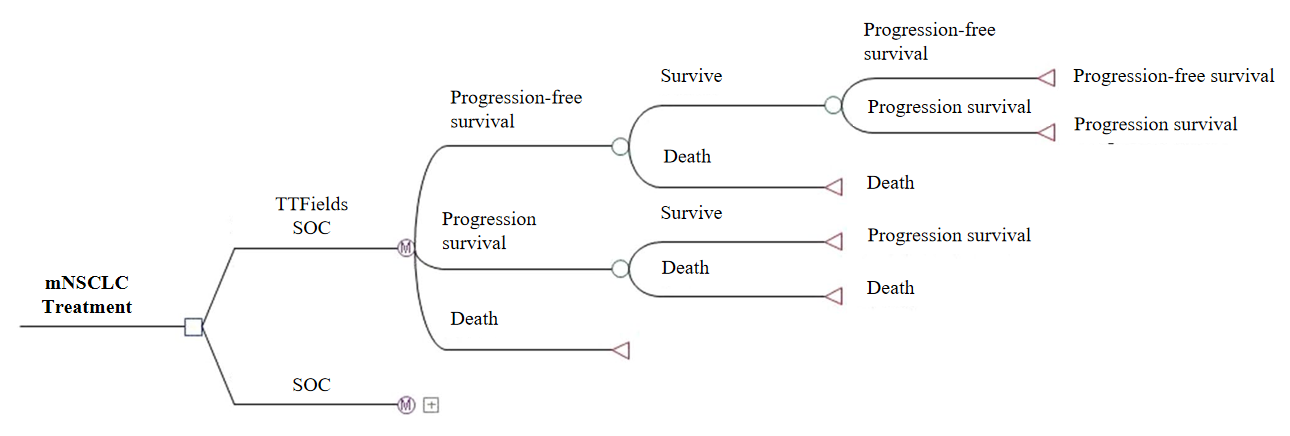


Abbreviation: TTFields, tumor treating field; SOC, standard of care; mNSCLC, metastatic non-small cell lung cancer; M, Markov.

**Figure S2. Kaplan-Meier Curve Fitting and Extrapolation.**

**
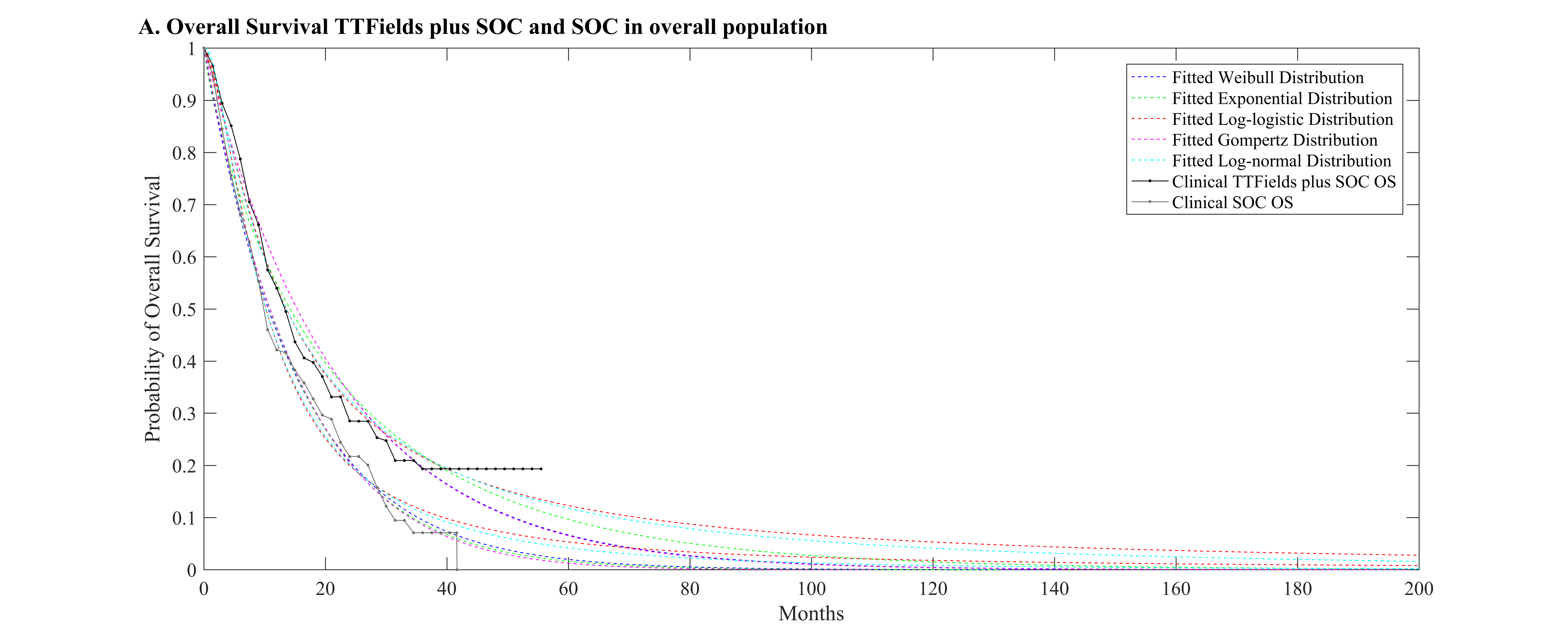
**

**
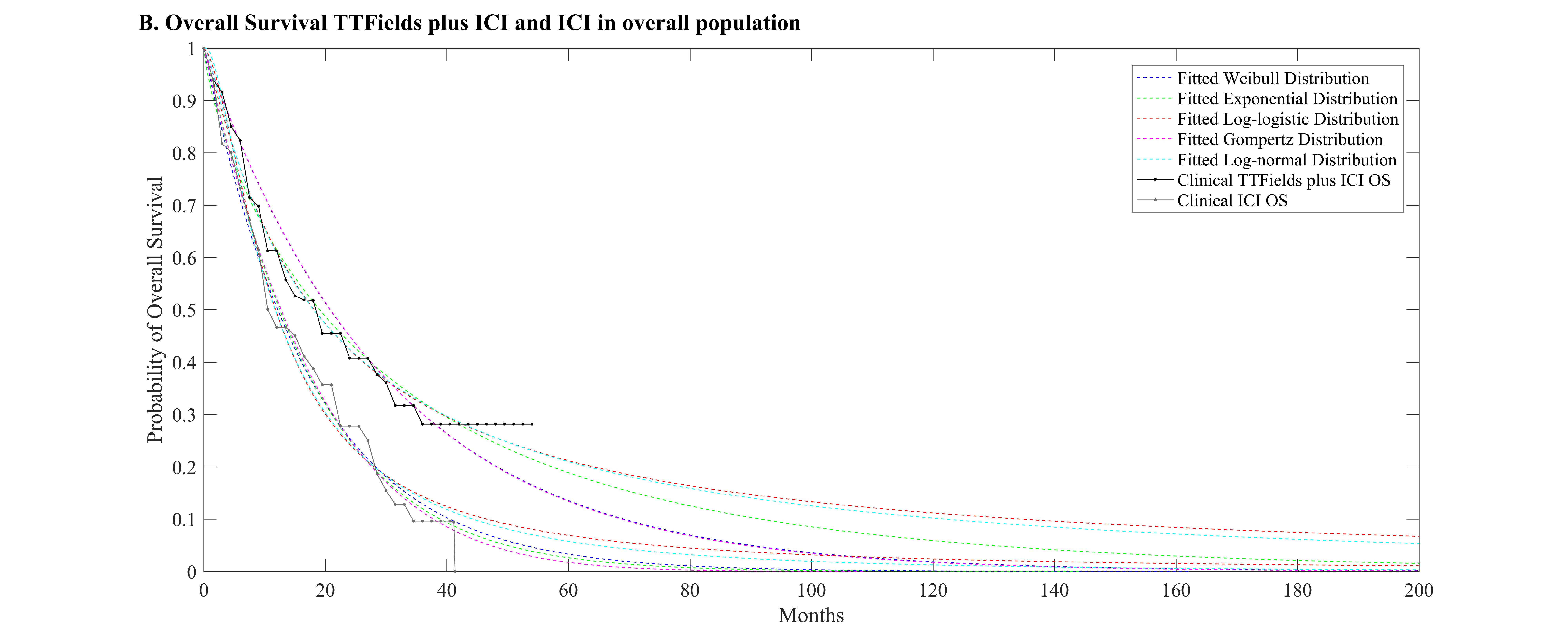

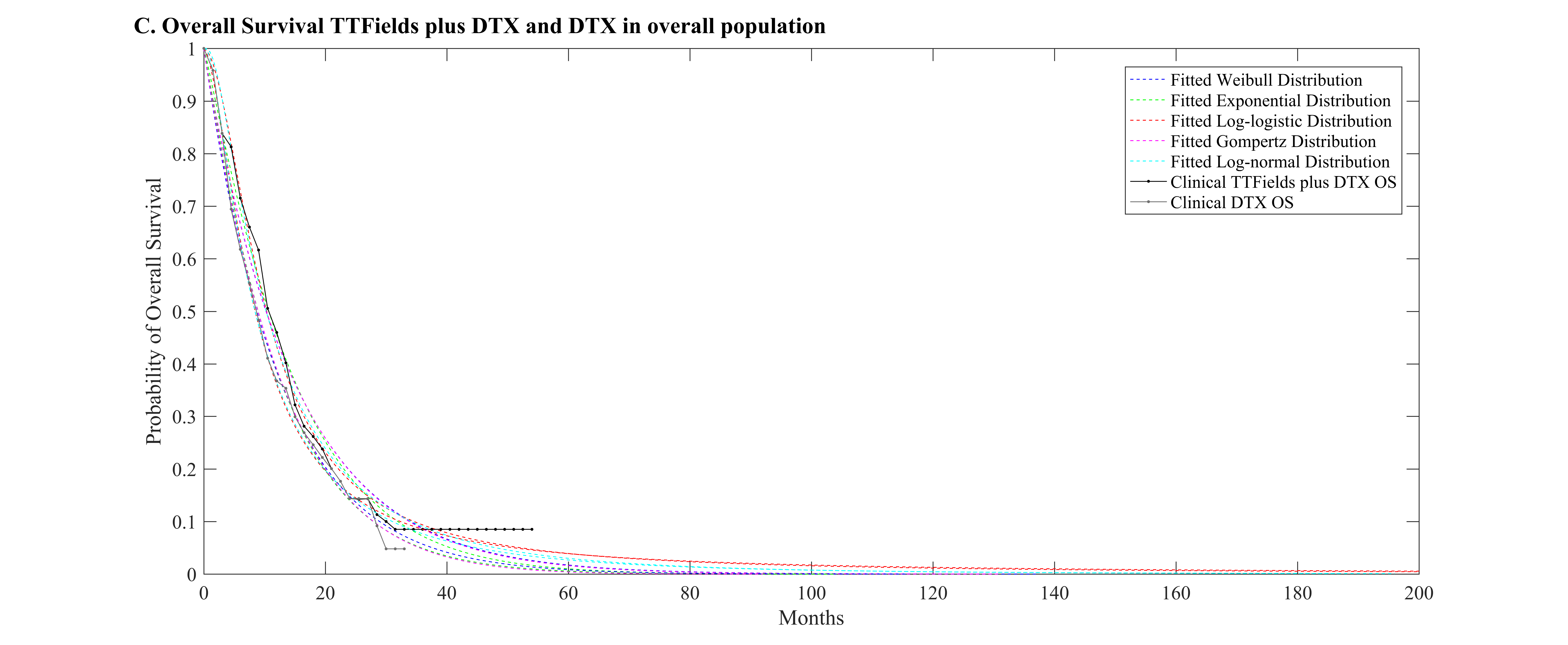
**

Abbreviation: TTFields, tumor treating field; SOC, standard of care; OS, overall survival; ICI, immune checkpoint inhibitor; DTX, docetaxel; PFS, progression-free survival.

**Figure S3.** **Probability Sensitivity Analysis Scatter Plot.**


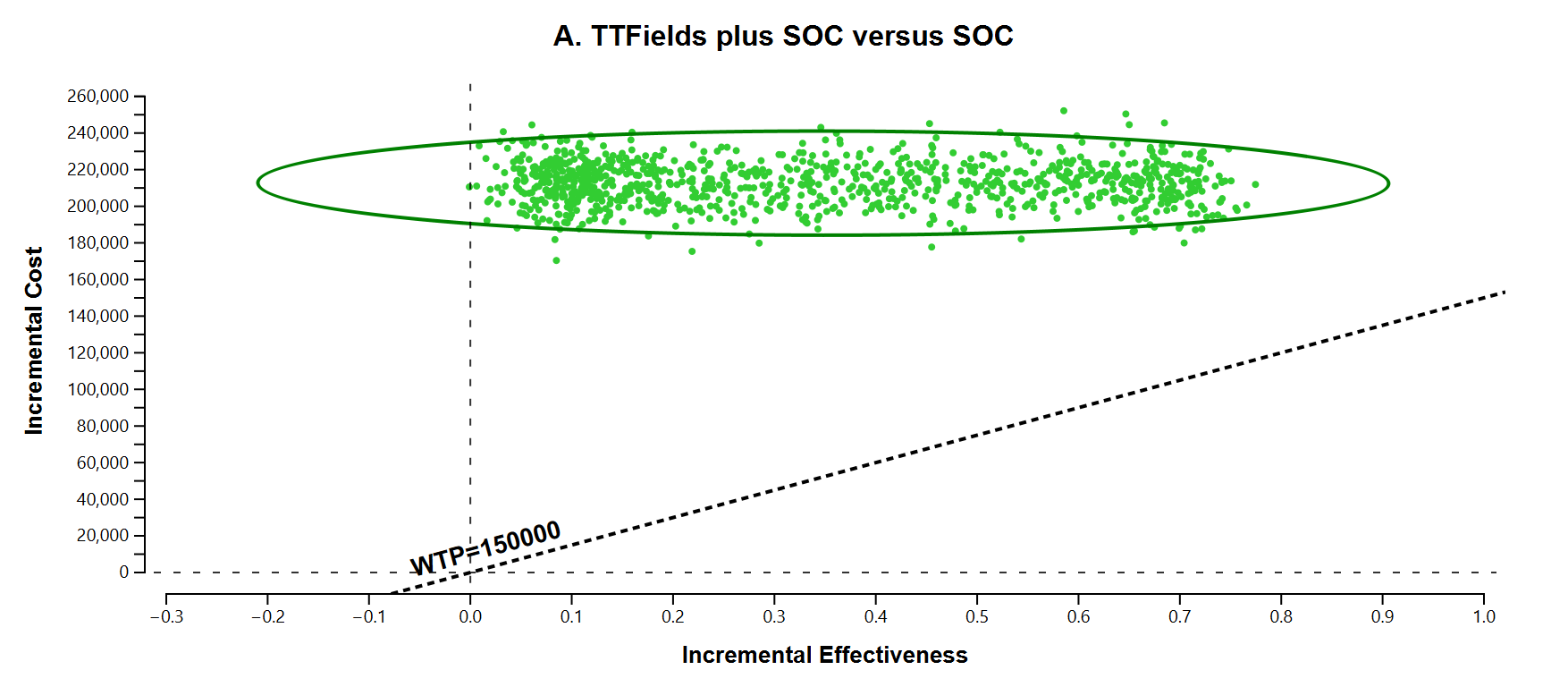


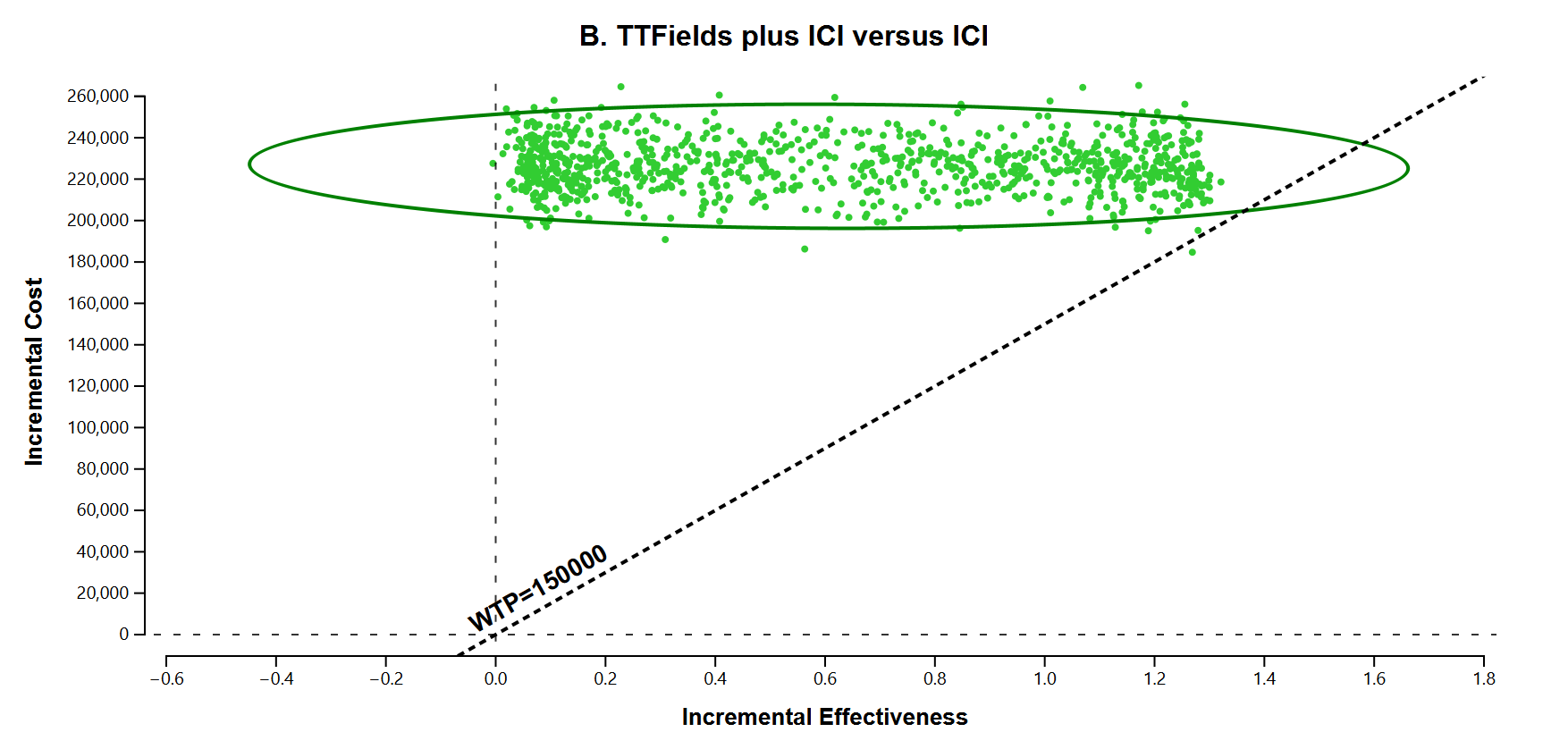


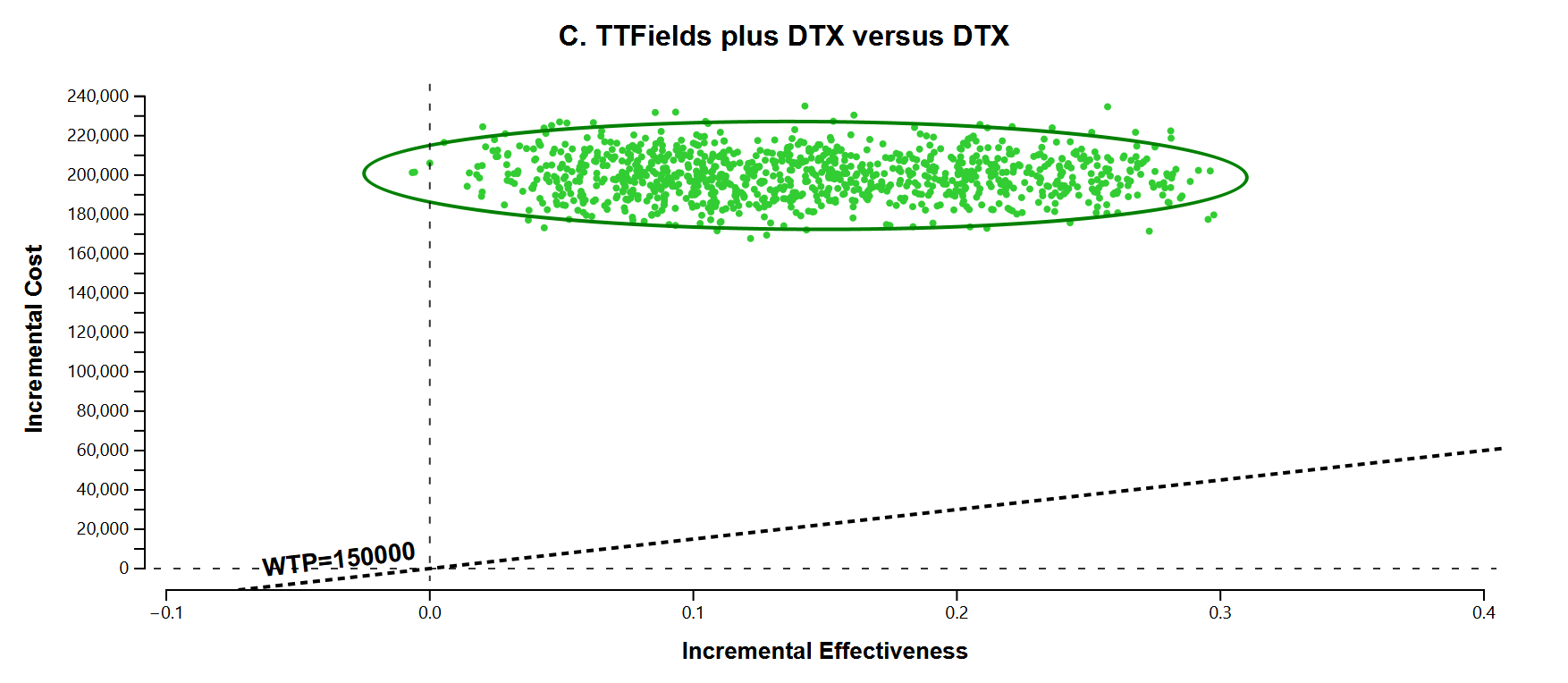


Abbreviation: TTFields, tumor treating field; SOC, standard of care; ICI, immune checkpoint inhibitor; DTX, docetaxel; WTP, willingness-to-pay.

Each point in the diagram represents a simulation result of 10,000 Monte Carlo simulations of TTFields plus SOC versus SOC in overall population (A), TTFields plus ICI versus ICI in overall population (B), TTFields plus DTX versus DTX in overall population (C). The ellipse represents the 95% CI and the dotted line represents WTP of $35,983/QALY. Below the dotted line is the probability of being cost-effective.

**Table S1. The CHEERS 2022 checklist.**

| **Section/item** | **Item No** | **Recommendation** | **Reported?** |
| --- | --- | --- | --- |
| **Title and abstract** | | | |
| Title | 1 | Identify the study as an economic evaluation and specify the interventions being compared. | Yes |
| Abstract | 2 | Provide a structured summary that highlights context, key methods, results, and alternative analyses. | Yes |
| **Introduction** | | | |
| Background and objectives | 3 | Give the context for the study, the study question, and its practical relevance for decision making in policy or practice. | Yes |
| **Methods** | | | |
| Health economic analysis plan | 4 | Indicate whether a health economic analysis plan was developed and where available. | Yes |
| Study population | 5 | Describe characteristics of the study population (such as age range, demographics, socioeconomic, or clinical characteristics). | Yes |
| Setting and location | 6 | Provide relevant contextual information that may influence findings. | Yes |
| Comparators | 7 | Describe the interventions or strategies being compared and why chosen. | Yes |
| Perspective | 8 | State the perspective(s) adopted by the study and why chosen. | Yes |
| Time horizon | 9 | State the time horizon for the study and why appropriate. | Yes |
| Discount rate | 10 | Report the discount rate(s) and reason chosen. | Yes |
| Selection of outcomes | 11 | Describe what outcomes were used as the measure(s) of benefit(s) and harm(s) | Yes |
| Measurement of outcomes | 12 | Describe how outcomes used to capture benefit(s) and harm(s) were measured. | Yes |
| Valuation of outcomes | 13 | Describe the population and methods used to measure and value outcomes. | Yes |
| Measurement and valuation of resources and costs | 14 | Describe how costs were valued. | Yes |
| Currency, price date, and conversion | 15 | Report the dates of the estimated resource quantities and unit costs, plus the currency and year of conversion. | Yes |
| Rationale and description of model | 16 | If modelling is used, describe in detail and why used. Report if the model is publicly available and where it can be accessed. | Yes |
| Analytics and assumptions | 17 | Describe any methods for analysing or statistically transforming data, any extrapolation methods, and approaches for validating any model used. | Yes |
| Characterizing heterogeneity | 18 | Describe any methods used for estimating how the results of the study vary for subgroups. | Yes |
| Characterizing distributional effects | 19 | Describe how impacts are distributed across different individuals or adjustments made to reflect priority populations. | Yes |
| Characterizing uncertainty | 20 | Describe methods to characterise any sources of uncertainty in the analysis. | Yes |
| Approach to engagement with patients and others affected by the study | 21 | Describe any approaches to engage patients or service recipients, the general public, communities, or stakeholders (such as clinicians or payers) in the design of the study. | Not applicable |
| **Results** | | | |
| Study parameters | 22 | Report all analytic inputs (such as values, ranges, references) including uncertainty or distributional assumptions. | Yes |
| Summary of main results | 23 | Report the mean values for the main categories of costs and outcomes of interest and summarise them in the most appropriate overall measure. | Yes |
| Effect of uncertainty | 24 | Describe how uncertainty about analytic judgments, inputs, or projections affect findings. Report the effect of choice of discount rate and time horizon, if applicable. | Yes |
| Effect of engagement with patients and others affected by the study | 25 | Report on any difference patient/service recipient, general public, community, or stakeholder involvement made to the approach or findings of the study. | Not  applicable |
| **Discussion** | | | |
| Study findings, limitations, generalizability, and current knowledge | 26 | Report key findings, limitations, ethical or equity considerations not captured, and how these could affect patients, policy, or practice. | Yes |
| **Other** | | | |
| Source of funding | 27 | Describe how the study was funded and any role of the funder in the identification, design, conduct, and reporting of the analysis | Yes |
| Conflicts of interest | 28 | Report authors conflicts of interest according to journal or International Committee of Medical Journal Editors requirements. | Yes |

*Reference:*

1. *Husereau D, Drummond M, Augustovski F, de Bekker-Grob E, Briggs AH, Carswell C, et al. Consolidated Health Economic Evaluation Reporting Standards 2022 (CHEERS 2022) Statement: Updated Reporting Guidance for Health Economic Evaluations. Value Health. 2022;25(1):3-9.*

**Table S2. Details of Treatment Strategy and Unit Costs.**

| **Treatment Strategy** | **Dose** | | **Time** | | **Unit costs, $** | |
| --- | --- | --- | --- | --- | --- | --- |
| TTFields | TTFields, 150 kHz | Administered 150 kHz ≥8 h/d; Monthly patch is required | | 9,355 | |  |
| ICI | Pembrolizumab, 200 mg | Administered intravenously every 3 weeks | | 16.7261 | |  |
|  | Nivolumab, 360 mg |  |  | 8.9891 | |  |
|  | Atezolizumab, 1200 mg |  |  | 2.1870 | |  |
| DTX | Docetaxel, 75 mg/m^2^ | Administered intravenously every 3 weeks | | 0.1582 | |  |

Abbreviation: TTFields, tumor treating field; ICI, immune checkpoint inhibitor; DTX, docetaxel.

**Table S3. Summary of Statistical Goodness-of-fit of K-M Curve.**

|  | **Exponential** | **Weibull** | **Gompertz** | **Log-logistic** | **Log-normal** |
| --- | --- | --- | --- | --- | --- |
| **TTFields plus SOC OS curve** | | | | | |
| AIC | 97.1900 | 94.0433 | 99.5567 | 92.7771 | 92.6948 |
| BIC | 98.8276 | 97.3185 | 102.8318 | 96.0523 | 95.9700 |
| **SOC OS curve** | | | | |  |
| AIC | 90.3785 | 86.9660 | 91.0705 | 85.6128 | 86.4767 |
| BIC | 93.1809 | 88.3672 | 93.8729 | 88.4152 | 89.2791 |
| **TTFields plus ICI OS curve** | | | | | |
| AIC | 68.6181 | 68.2455 | 70.6793 | 67.9479 | 67.8326 |
| BIC | 71.4673 | 71.2291 | 73.9012 | 71.1697 | 71.0545 |
| **ICI OS curve** | | | | |  |
| AIC | 79.3149 | 77.9770 | 80.3521 | 76.0272 | 76.5535 |
| BIC | 82.1173 | 79.3782 | 83.1545 | 78.8296 | 79.3559 |
| **TTFields plus DTX OS curve** | | | | | |
| AIC | 148.7209 | 137.2111 | 140.1499 | 132.0763 | 134.6791 |
| BIC | 151.9428 | 138.8220 | 143.3717 | 135.2981 | 137.9009 |
| **DTX OS curve** |  |  |  |  |  |
| AIC | 65.6254 | 64.3809 | 65.5409 | 62.7606 | 63.2218 |
| BIC | 67.8964 | 65.5164 | 67.8119 | 65.0316 | 65.4928 |
| **TTFields plus SOC PFS curve** | | | | | |
| AIC | 34.7522 | 34.3519 | 36.3996 | 34.1839 | 34.2437 |
| BIC | 35.8821 | 35.8069 | 37.5295 | 35.3138 | 35.3736 |
| **SOC PFS curve** |  |  |  |  |  |
| AIC | 39.3754 | 38.3302 | 41.4542 | 37.5561 | 37.7022 |
| BIC | 39.9404 | 39.4601 | 42.5841 | 38.6860 | 38.8321 |

Abbreviation: TTFields, tumor treating field; SOC, standard of care; OS, overall survival; PFS, progression-free survival; AIC, Akaike’s information criterion; BIC, Bayesian information criterion; ICI, immune checkpoint inhibitor; DTX, docetaxel.

As for the ten curves listed in the table, the log-normal and log-logistic distribution had the lowest AIC and BIC. While the AIC and BIC tests is important to determine which models fit the observed data best, it does not tell us anything about how suitable a parametric model is for the time period beyond the final trial follow-up. They described the internal validity of fitted models, but not their external validity. The Log-logistic and lognormal models can incorporate non-monotonic hazards but typically have long tails due to a reducing hazard as time increases after a certain point. Actually, the visual fits of the eight curves (Figure S2) showed that log-normal and log-logistic distribution extended tail, which would likely overestimate OS and PFS in the long term based on clinical experts’ opinion.

Weibull distributions are flexible and wildly used in cancer survival analyses. Therefore, the Weibull distributions was likely to be the most reasonable parametric survival model besides the log-logistic and log-normal distribution.

*Reference:*

1. *Latimer NR. Survival analysis for economic evaluations alongside clinical trials--extrapolation with patient-level data: inconsistencies, limitations, and a practical guide. Med Decis Making. 2013 Aug;33(6):743-54. doi: 10.1177/0272989X12472398.*
2. *Han J, Tian K, Yang J, Gong Y. Durvalumab vs placebo consolidation therapy after chemoradiotherapy in stage III non-small-cell lung cancer: An updated PACIFIC trial-based cost-effectiveness analysis. Lung Cancer. 2020 Aug;146:42-49. doi: 10.1016/j.lungcan.2020.05.011.*

**Table S4. Results of Subgroup Analyses.**

| **Subgroup** | **OS HR (95% CI)** | **Incremental cost, $** | **Incremental QALYs** | **ICER, $/QALY** | **Cost-effectiveness probability of TTFields plus SOC at WTP, %** |
| --- | --- | --- | --- | --- | --- |
|  |  |  |  |  | **$35,983/QALY** |
| **Pathological type** |  |  |  |  |  |
| SCC | 0.67 (0.44 to 1.01) | 72,433 (71,390 to 85,710) | 0.26 (0.04 to 0.44) | 273,741 | 0% |
| NSCC | 0.80 (0.54 to 1.16) | 73,484 (62,311 to 81,441) | 0.18 (-0.16 to 0.56) | 410,594 | 0% |

Abbreviation: TTFields, tumor treating field; SOC, standard of care; SCC, squamous cell carcinoma; NSCC, non-squamous cell carcinoma.

**Table S5. Discounting and Cost-Effectiveness Results.**

| **Cost of TTFields** | **Incremental cost, $** | **Incremental benefits, QALYs** | **ICER, $/QALY** | **Comments** |
| --- | --- | --- | --- | --- |
| **TTFields plus SOC versus SOC** | | | | |
| 50% cost | 38,736 | 0.26 | 147,546 | Not cost-effective |
| 10% cost | 9,974 | 0.26 | 37,991 | Not cost-effective |
| 5% cost | 6,379 | 0.26 | 24,296 | Cost-effective |
| **TTFields plus ICI versus ICI** | | | | |
| 50% cost | 40,940 | 0.42 | 98,233 | Not cost-effective |
| 10% cost | 11,200 | 0.42 | 26,873 | Cost-effective |
| 5% cost | 7,482 | 0.42 | 17,953 | Cost-effective |
| **TTFields plus DTX versus DTX** | | | | |
| 50% cost | 36,690 | 0.13 | 281,133 | Not cost-effective |
| 10% cost | 8,996 | 0.13 | 68,930 | Not cost-effective |
| 5% cost | 5,534 | 0.13 | 42,405 | Not cost-effective |

Abbreviation: ICER, incremental cost-effectiveness ratio; LY, life-year; QALY, quality-adjusted life-year; SOC, the standard-of-care; ICI, immune checkpoint inhibitor; DTX, docetaxel.

.
